# Tonic immobility in terrestrial isopods: intraspecific and interspecific variability

**DOI:** 10.3897/zookeys.176.2355

**Published:** 2012-03-20

**Authors:** Aline Ferreira Quadros, Priscila Silva Bugs, Paula Beatriz Araujo

**Affiliations:** 1 Programa de Pós-Graduação em Biologia Animal. Departamento de Zoologia, IB, Universidade Federal do Rio Grande do Sul, Av. Bento Gonçalves, 9500, prédio 43435, CEP 91501-970, Porto Alegre, Rio Grande do Sul, Brasil

**Keywords:** Death-feigning, anti-predator strategies, Crinocheta

## Abstract

Many arthropods, including terrestrial isopods, are capable of entering a state of tonic immobility upon a mechanical disturbance. Here we compare the responses to mechanical stimulation in three terrestrial isopods *Balloniscus glaber*, *Balloniscus sellowii* and *Porcellio dilatatus*. We applied three stimuli in a random order and recorded whether each individual was responsive (i.e. showed tonic immobility) or not and the duration of the response. In another trial we related the time needed to elicit tonic immobility and the duration of response of each individual. *Balloniscus sellowii* was the least responsive species and *Porcellio dilatatus* was the most, with 23% and 89% of the tested individuals, respectively, being responsive. Smaller *Balloniscus sellowii* were more responsive than larger individuals. *Porcellio dilatatus* responded more promptly than the *Balloniscus* spp. but it showed the shortest response. Neither sex, size nor the type of stimulus explained the variability found in the duration of tonic immobility. These results reveal a large variability in tonic immobility behavior, even between closely related species, which seems to reflect a species-specific response to predators with different foraging modes.

## Introduction

To be successful in avoiding their predators, preys engage in a multitude of behaviors, such as the construction of shelters (Manicom et al. 2008), decrease in activity and change in activity period according to their predator’s (Sakamoto et al. 2006), change of foraging sites to decrease predator encounter chances (Lima and Dill 1990) and even foraging in sites that will allow a better chance of escape (Wirsing et al. 2007). However, predators have their strategies too, which often leads them to their prey. Once in close contact with a predator, prey may engage in a second class of anti-predator strategies, i.e. to avoid being captured and/or being consumed. Amongst this class we find behaviors such as autotomy, release of chemical substances and tonic immobility (Langerhans 2007).

Tonic immobility is a state of reversible physical immobility and muscle hypertonicity, during which the organism lack responsiveness to external stimulation (Gallup 1974). It is a widespread form of passive anti-predator behavior employed by a variety of animals such as reptiles (Santos et al. 2010), harvestmen (Machado and Pomini 2008), orthopterans (Nishino and Sakai 1996, Faisal and Matheson 2001), coleopterans (Miyatake et al. 2004), hymenopterans (King and Leaich 2006) and crustaceans (Holmes 1903, Saxena 1957, Bergey and Weis 2006, Scarton et al. 2009). Tonic immobility is often called thanatosis or death-feigning, but these terms may be misleading in some cases where animals engaging in tonic immobility often assume different positions than dead animals (see Honma et al. 2006). Tonic immobility has been intensely addressed experimentally, especially in arthropods (Prohammer and Wade 1981, Honma et al. 2006, Miyatake et al. 2009, Nakayama and Miyatake 2010). It has been show to function as a defense mechanism and have an adaptive significance in several situations. One is when immobility physically impedes consumption. Honma et al. (2006) demonstrated that the consumption of the grasshopper *Criotettix japonicus* (Haan) by its frog predator *Rana nigromaculata* Hallowell was reduced because the posture assumed during tonic immobility enlarged the grasshopper’s functional size and this was effective against the gape-limited predator (Honma et al. 2006). Other situations are when predators lose interest in unmoving prey (Miyatake et al. 2004) and when feigners are less likely to be preyed when in the presence of non-feigners (Miyatake et al. 2009). One common characteristic that emerges from these studies is that there is often a high intraspecific variability in tonic immobility behavior. This variability manifests both in relation to the responsiveness of the individuals and in the duration of their responses. It has been shown that both characteristics have a genetic basis (Prohammer and Wade 1981, Miyatake et al. 2004).

Amongst crustaceans, tonic immobility has been observed in crabs and terrestrial amphipods and isopods. Terrestrial isopods (Oniscidea) are preyed upon by a large variety of animals: spiders (Řezáč and Pekár 2007), chilopods (Sunderland and Sutton 1980), opiliones (Santos and Gnaspini 2002), ants (Dejean 1997), land flatworms (Prasniski and Leal-Zanchet 2009), amphibians and reptiles (Vitt et al. 2000, Van Sluys et al. 2001), among others. These predators employ a variety of prey-seeking and prey-handling behaviors. Naturally, terrestrial isopods have responded to this predation pressure by developing a variety of anti-predator strategies, which include a combination of behavioral and morphological traits (Schmalfuss 1984) and also a form of chemical defense which is unique among crustaceans (Gorvett 1956, Deslippe et al. 1996). According to behavioral and morphological traits related to predator avoidance, isopod species can be grouped into ecomorphological groups, as proposed by Schmalfuss (1984): The “runners” are narrow, have long and strong pereopods adapted to run and escape fast when they are uncovered or disturbed. The “clingers”, on the other hand, have broad tergites, short pereopods, and flattened bodies and when disturbed, tend to remain motionless and tightly attached to the substrate. The “rollers” are capable of entering a state of tonic immobility when disturbed, forming a near perfect or a perfect ball which encloses the pereopods and pleopods and hides the ventral surface of the animal. The “spiny forms” are also conglobating forms that have, in addition, conspicuous dorsal spiny protuberances (Schmalfuss 1984).

The conglobation ability of “rollers” and “spiny forms” is, for sure, a well-known example of tonic immobility in response to disturbance. As pointed by Saxena (1957) when referring to the roller *Armadillidium vulgare* Latreille “*a light pressure on the ventral side and sometimes a fall from a height of 2 or 3 inches will bring about this motionless state and induce the characteristic roll up into a ball like shape*”. However, many other species of different ecomorphological groups, such as clingers (Schmalfuss 1984) and runners (Sokolowicz et al. 2008) are also capable of entering a state of tonic immobility upon disturbance. In such groups, tonic immobility involves the contraction of the body to form a comma-like shape and the contraction and folding of the legs towards the ventral side while holding the antennae folded or extended backwards and pressed against the dorsal contour of the first pereonites (pers. obs.). Although tonic immobility is a widespread response in terrestrial isopods, it has not been addressed experimentally, except for the work of Saxena (1957). Here we compare the responses to mechanical stimuli of three species belonging to the clinger ecomorphological group. Two are close-related species: *Balloniscus glaber* Araujo & Zardo and *Balloniscus sellowii* (Brandt) (Balloniscidae) and the other is a Porcellionidae, *Porcellio dilatatus* Brandt. We ask the following questions: (1) Do the species differ in responsiveness to tonic immobility-inducing stimuli? (2) Does the responsiveness depend upon sex, size or stimulus? (3) Is the duration of tonic immobility influenced by sex, size or type of stimulus, and does it differs between species? (4) Is the duration of tonic immobility related to the time needed to elicit a response?

## Methods

### Species sampling and laboratory conditions

**Figure 1.**
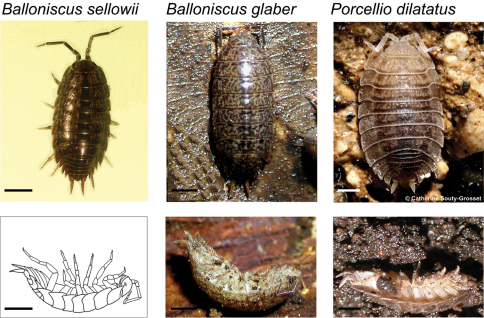
Terrestrial isopods studied in dorsal view: *Balloniscus sellowii*, *Balloniscus glaber* (Balloniscidae) and *Porcellio dilatatus* (Porcellionidae) (top) and their respective postures during tonic immobility (bottom). For *Balloniscus sellowii* a drawing made from a photograph is presented. Bars = 2 mm.

Leaf litter samples containing *Balloniscus glaber*, *Balloniscus sellowii* and *Porcellio dilatatus* (Fig. 1) were collected in urban areas and vicinities of the campus of Universidade Federal do Rio Grande do Sul, in Porto Alegre, in the south of Brazil. *Balloniscus glaber* were captured in July 2008, and *Balloniscus sellowii* and *Porcellio scaber*, in July and November 2009, respectively.

In the laboratory, we randomly pick 60 intermoult individuals (near 1:1 sex ratio) of each species and put them individually in Petri dishes (9 cm diameter)containing moist soil, food (decayed leaves) and plastic black shelters. They were left undisturbed for 72 h prior to the trials. Ovigerous females or females with an empty marsupium were not used in the trials. The isopods were maintained side by side in a large shelve, about 1.30 m height, illuminated by phosphorescent light, in a room with temperature of 20°C and a 12:12 photoperiod. To minimize manipulation prior the trials, the individuals were measured (cephalothorax width) only at the end of the experiments.

### Types of stimuli

We choose three different stimuli to be applied to the isopods: touch, squeeze and drop. These stimuli are known to elicit tonic immobility as they frequently did so when we are handling isopods of various species in the laboratory, and they could mimic the mode of action of different predators. The stimulus “touch” consisted of repeatedly pinching the isopod’s body laterally with the tip of a metal forceps and gently pushing it. When inspecting soil and litter samples, it is very common to observe tonic immobility in the isopods after touching/pushing then (pers. obs.). The stimulus “squeeze” consisted of grabbing the isopod with a forceps and slightly squeezing it, in an attempt to simulate the bite of an ant. The “drop” stimulus consisted of grabbing the isopod with a forceps, lifting it approx. 10 cm and then letting it drop in the dish. Here we were trying to mimic a larger predator, such as a small bird or lizard, letting the isopod to fall after grabbing it. The experiments that will be described below were conducted always by two observers, and the observer that applied the stimuli to the isopods was the same throughout all the trials. Care was taken to apply the stimuli and not causing injuries to the isopods. In fact, seven *Porcellio dilatatus* and one *Balloniscus sellowii* died during the acclimation period but there was no mortality during the experiments.

### Experiment 1

To answer questions 1, 2 and 3 we applied the three different stimuli described above to each individual in a random sequence. Each species was tested on a separate day, and all individuals of the same species were tested on the same day. Before starting the trials, the shelters and leaves were removed from all petri dishes to allow a proper observation of isopod behavior.

We started the trial by randomly picking a number corresponding to an individual and a number corresponding to a stimulus to be applied to that individual (e.g. 1 for touch, 2 for squeeze, 3 for drop). Then, we removed the lid of the petri dish and applied the stimulus to the isopod. The stimulus was repeated up to 3× in case of “squeeze” or “drop” or 5× in case of “touch”. If the stimulus did not elicited tonic immobility, the individual was considered non-responsive. If the individual responded, i.e. showed the characteristic posture of tonic immobility (see Fig. 1), the duration of tonic immobility was recorded with a stopwatch. The end of the response was when the individual showed any slight movement, which usually began with a movement of the antenna. The lid was placed again on the dish and another number was randomly picked. These procedures were repeated until all individuals have been subject to the three stimuli.

### Experiment 2

To answer question 4 we conducted a second experiment, on the day after experiment 1, with the same individuals. The order of the individual was defined randomly as in the experiment 1. We removed the lid of the dish and stressed the isopod with the tip of a forceps for up to 30 seconds, i.e. applying only the “touch” stimulus. If during this period the stimulus did not elicit tonic immobility, the individual was considered non-responsive. If the isopod responded, it was recorded both the time it took to respond (to enter the posture) and the duration of response.

### Analyses

Proportion data from experiment 1 was compared with a G test of independence (for two samples) or a chi-square test (one sample). The relationship between individual size and its responsiveness was investigated with a logistic regression. To answer question 3, the differences in the duration of tonic immobility among species and stimuli was tested with ANOVA and Tukey test. Also for each species the relationship between duration and size was tested with ANCOVA using sex as the co-variable. Finally, to answer question 4, a linear regression was applied to verify if there was relationship between the time to elicit tonic immobility and duration of response (experiment 2). For all tests the duration of response was log-transformed. All tests were made using the R^©^ Software v. 2.13.0 (The R Foundation for Statistical Computing 2011).

## Results

### Experiment 1

#### Do the species differ in responsiveness?

**Figure 2.**
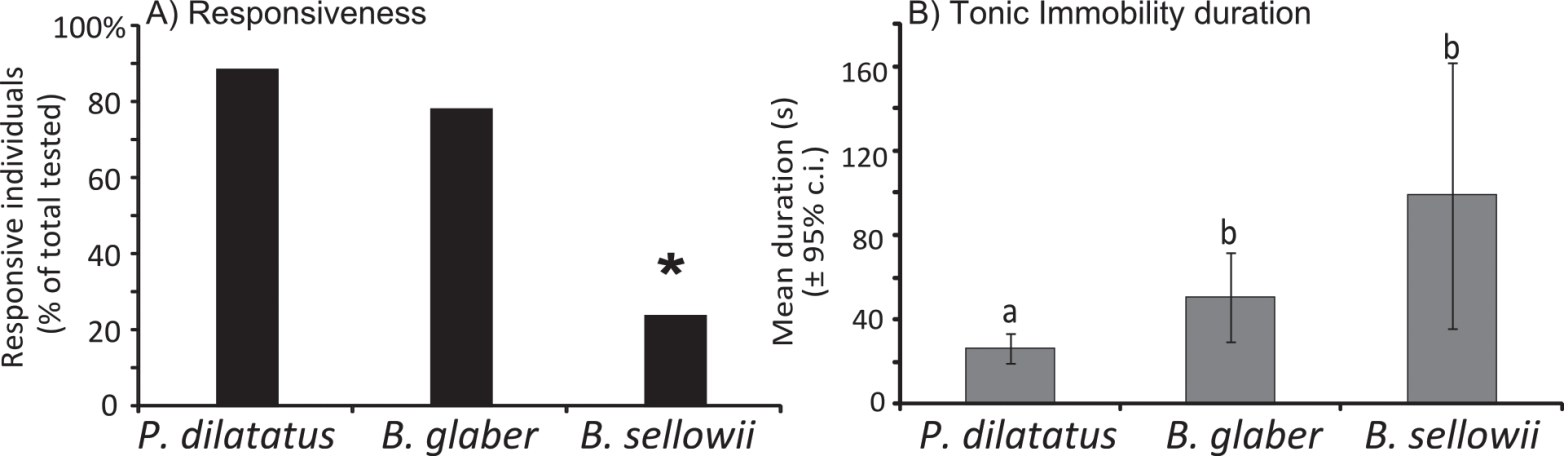
Responsiveness and tonic immobility duration in terrestrial isopods. **A** Percentage of responsive individuals in the three species tested. The * indicates a significant difference between species, after a G-test. **B** Mean tonic immobility duration in seconds for each terrestrial isopod (considering all stimuli pooled) in experiment 1. Different letters indicate significant differences, after ANOVA and Tukey test.

*Porcellio dilatatus* and *Balloniscus glaber* differed remarkably from *Balloniscus sellowii* (*G*=62.26; *p*<0.0001) (Fig. 2A): *Porcellio dilatatus* and *Balloniscus glaber* were the most responsive species and did not differ from each other; 89% and 78% of their individuals, respectively, were responsive to at least one of the stimuli applied; 51% of the responsive *Porcellio dilatatus* individuals were responsive to all three stimuli applied, while only 12% of *Balloniscus glaber* individuals’ were so. *Balloniscus sellowii* differed from both species in that only 23% of the tested individuals were responsive to at least one stimulus and none responded to all three stimuli (Fig. 2A).

#### Does the responsiveness change according to the sex, stimuli and size?

**Figure 3.**
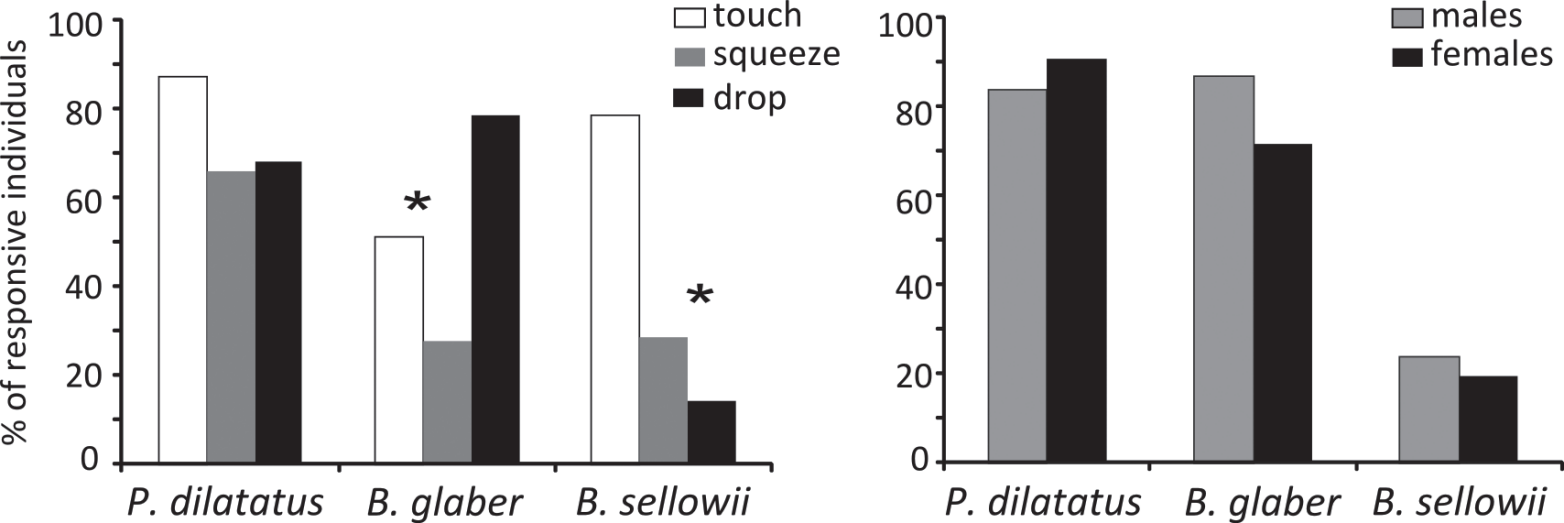
**A** Percentage of responsive individuals to each specific stimulus, in relation to the total number of responsive individuals of each species. **B** Percentage of responsive males and females in relation to the total number of males and females of each species tested. The * indicates a significant difference between stimuli, after a χ^2^ test.

Regarding the different stimuli, *Porcellio dilatatus* was responsive to the three stimuli equally (χ^2^=1.75; *p*=0.417), whereas *Balloniscus glaber* was more responsive to “drop” (χ^2^=11.703; *p*=0.003) and *Balloniscus sellowii* was more responsive to “touch” (Fig. 3A) (χ^2^=7.882; *p*=0.019). The proportion of responsive males and females did not differ in all species (*Porcellio dilatatus* χ^2^=0.111; *p*=0.73; *Balloniscus glaber* χ^2^=0.812; *p*=0.36; *Balloniscus sellowii* χ^2^=0.008; *p*=0.92) (Fig. 3B).

**Figure 4.**
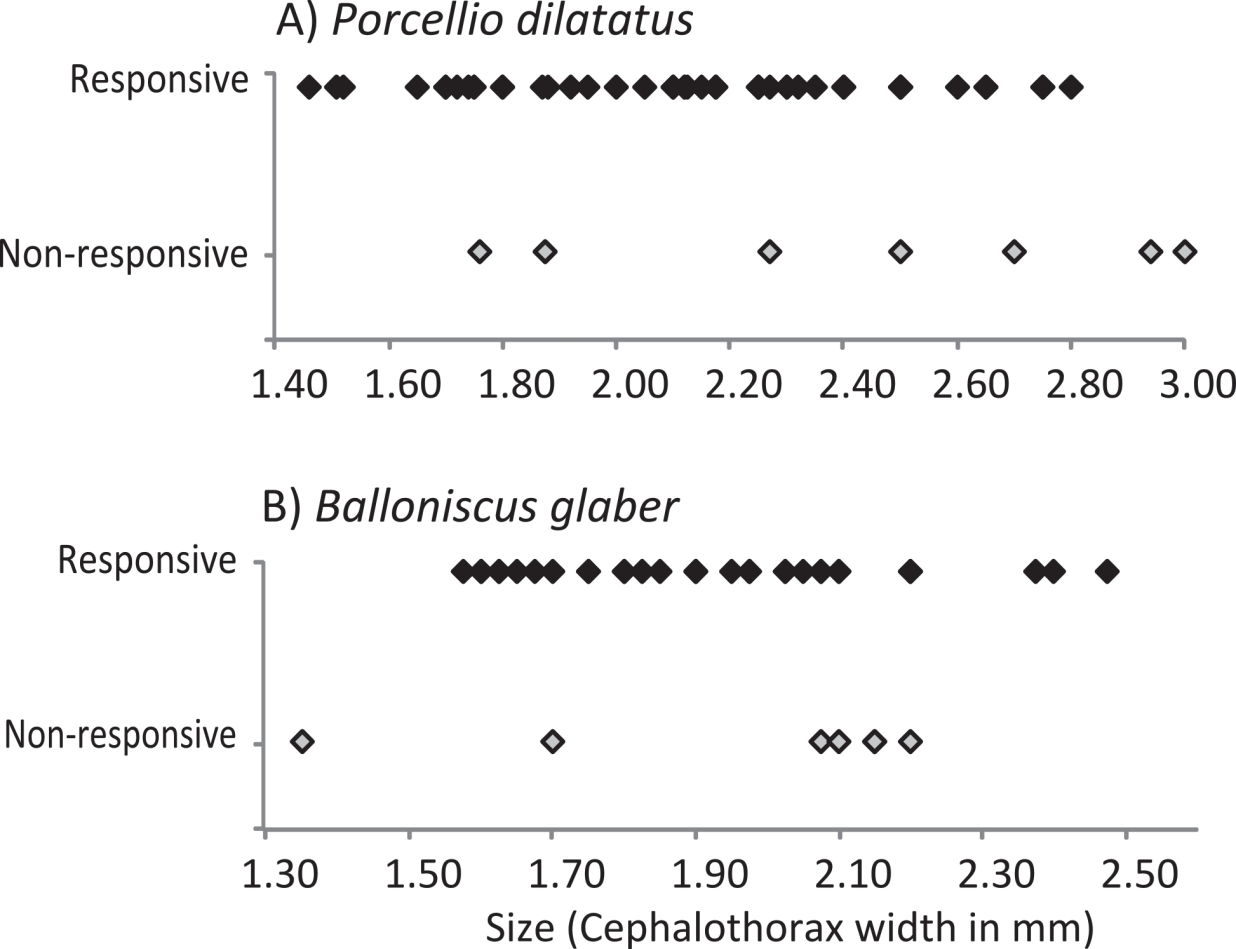
Size and response to the stimuli in **A** *Porcellio dilatatus* and **B** *Balloniscus glaber*. Responsive individuals are represented with black marks and non-responsive individuals with grey marks.

**Figure 5.**
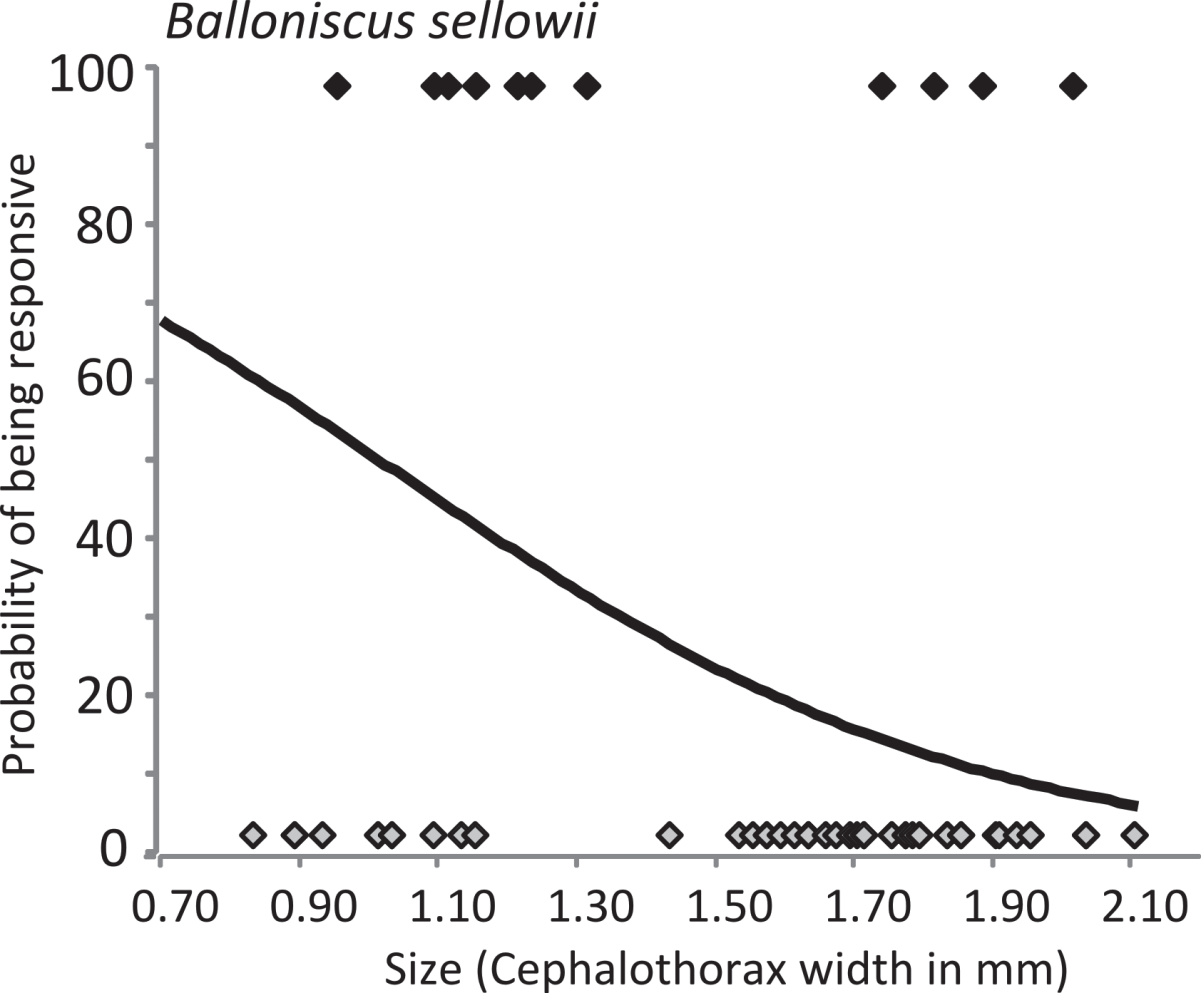
Responsiveness of *Balloniscus sellowii* individuals in relation to size. The line models the probability of being responsive according to the individual size (after a logistic regression). The black and grey symbols show the responsive and non-responsive individuals, respectively.

In relation to size, an interesting trend was observed. In *Porcellio dilatatus* and *Balloniscus glaber*, responsiveness was independent of individual size. As mentioned above, most individuals of these species were responsive (Fig. 4). In *Balloniscus sellowii*, however, there were intraspecific differences in responsiveness: a fitted logistic regression indicated that the probability of being responsive is higher in younger individuals and decreased with size (=age) (Fig. 5) (Logistic regression: Model intercept=3.254; s.e.=1.39; *p*=0.019; Size (factor) = -2.821; s.e.= 0.951; *p*=0.003; AIC=60.21).

#### Is the duration of tonic immobility influenced by the species, stimulus, sex, or size?

The duration of tonic immobility was highly variable in all trials: it ranged from a few seconds up to 3 min in *Porcellio dilatatus*, 7 min in *Balloniscus sellowii* and up to 12 min in *Balloniscus glaber*. The duration of response differed between species (Fig. 2B), but the effect of the different stimuli was not significant (Table 1). *Porcellio dilatatus* remained in tonic immobility for a shorter time interval than the two *Balloniscus* species (Fig. 2B).

**Table 1.** Differences in the duration of tonic immobility depending on the species and different stimuli.

	**ANOVA results**				
Factors	d.f.	SS	MS	*F*	*p*
Species	2	8.91	4.45	13.36	*p*<0.001
Stimuli	2	0.93	0.46	1.39	*p*=0.250
Residuals	190	63.34	0.33		

Using ANCOVA we verified that neither sex nor size of the isopods explained the variation found in the duration of tonic immobility (Table 2).

**Table 2.** Relationship between the duration of tonic immobility and individual size (cephalothorax width in mm) and sex (co-variable).

ANCOVA results				
Factors	d.f.	SS	*F*	*p*
	*Porcellio dilatatus*			
Size	1	0.143	0.587	0.44
Sex	1	0.836	3.426	0.07
Size:Sex	1	0.147	0.602	0.44
Residuals	37	9.035		
	*Balloniscus glaber*			
Size	1	0.052	0.226	0.63
Sex	1	0.287	1.248	0.27
Size:Sex	1	0.019	0.085	0.77
Residuals	27	6.215		
	*Balloniscus sellowii*			
Size	1	0.366	1.127	0.30
Sex	1	0.514	1.582	0.22
Size:Sex	1	0.516	1.583	0.22
Residuals	16	5.205		

### Experiment 2

#### Is the duration of tonic immobility related to the time needed to elicit a response?

**Figure 6.**
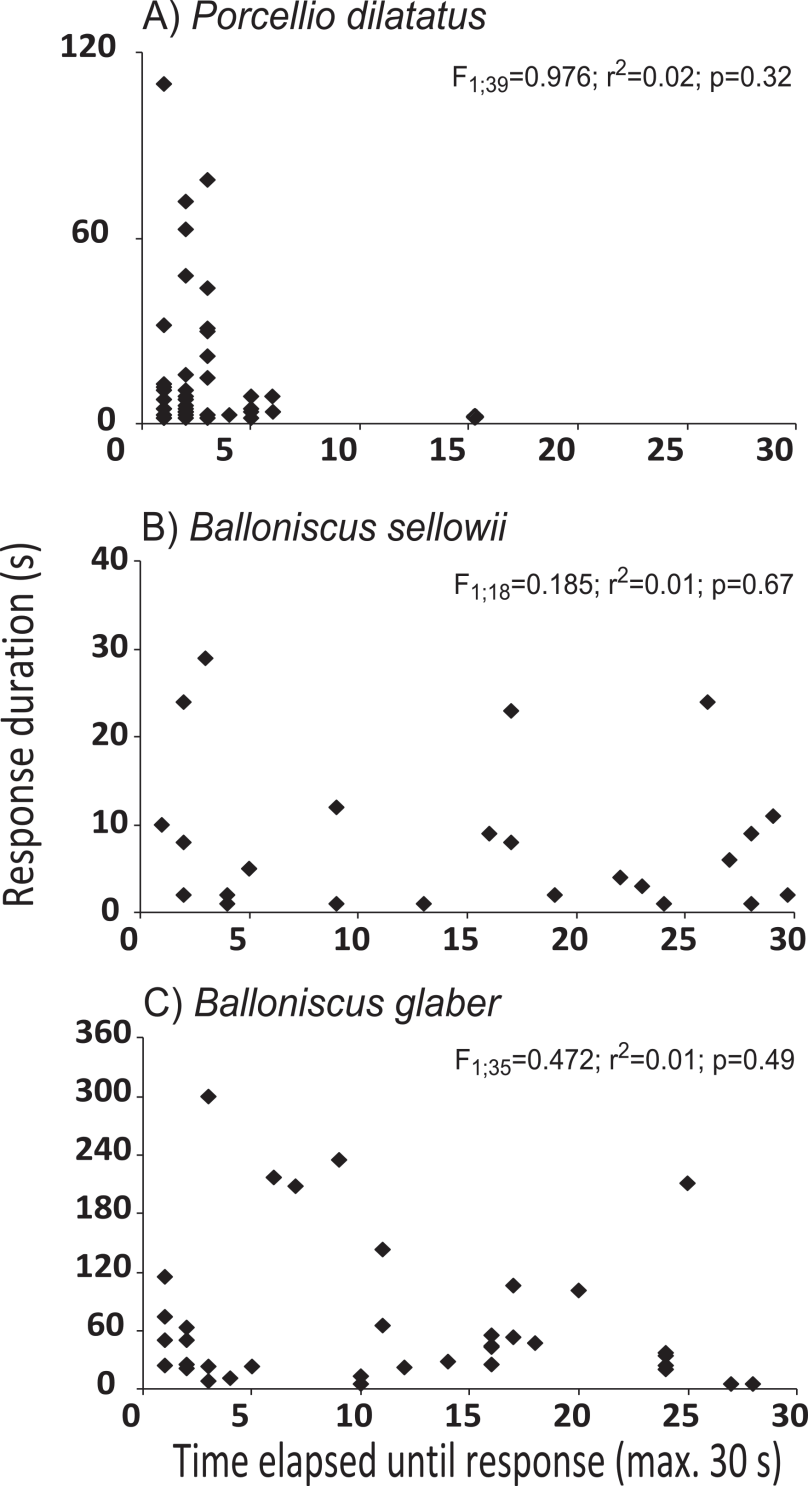
Relationship between the time elapsed until the beginning of tonic immobility and the duration of response, for responsive individuals in experiment 2. The values indicate the results of the linear regression analysis.

In this experiment, 77% *Porcellio dilatatus*, 65% *Balloniscus glaber* and 34% *Balloniscus sellowii* individuals responded to the stimulus within the 30 seconds. Upon visual inspection of data in Fig. 6 it can be seen that for none of the species there was a relationship between the time elapsed until tonic immobility and the duration of the response, which was confirmed with the linear regression analysis (see Fig. 6). In the case of both *Balloniscus* species there were individuals that responded promptly (in less than 5 s) or took more than 20 s to respond and remained in tonic immobility for short time intervals (less than 10 sec) and long time intervals (4 to 5 min). In all species tonic immobility duration was highly variable among individuals and it was independent of the time that each individual took to respond (Fig. 6). However, it can be noted a different pattern of response in *Porcellio dilatatus*: 99% of the responsive individuals responded within 7 s of stimulation (Fig. 6).

## Discussion

In this study we used two intrinsic factors (sex and size of the individuals) and one extrinsic factor (different stimuli) to try to explain the intraspecific variability found with respect to the responsiveness and duration of the tonic immobility showed by terrestrial isopods. We found an influence of the size in the responsiveness of *Balloniscus sellowii* and an influence of the type of stimulus in both *Balloniscus* species. Regarding the duration of tonic immobility, none of the factors explained the variability exhibited by the individuals. These findings are discussed below in more detail.

Males and females of different species are known to differ in responsiveness and duration of tonic immobility. For instance, in the beetle *Callosobruchus maculatus* (Fabricius) the females had a significantly higher frequency and longer duration of tonic immobility than males (Myatake et al. 2008a). In the freshwater crab *Trichodactylus panoplus* von Martens females stayed immobile for longer time than males (Scarton et al. 2009). In the terrestrial isopods studied here there was no indication of any sex-related differences in tonic immobility behavior, and maybe this indicates that both males and females are exposed to the same predators.

Upon encounter with a predator, a prey may run or enter tonic immobility, but it cannot adopt both strategies at the same time (Ohno and Miyatake 2007). In fact, there are many examples in the literature showing that prey specializes in one type of strategy at the expense of the other (Miyatake 2001a). King and Leaich (2006) found a negative relationship between tonic immobility and locomotor activity in the parasitoid wasp *Nasonia vitripennis* Walker. Also, a negative genetic correlation between tonic immobility intensity and locomotor activity (Nakayama and Miyatake 2010) was demonstrated in the beetle *Callosobruchus chinensis* (L.), both for field populations and populations artificially selected for either tonic immobility intensity or flying ability (Ohno and Miyatake 2007). Here we observe that, in contrast to the other species, *Balloniscus sellowii* does not engage in tonic immobility very often, and their smaller (= younger) individuals were more likely to do so than the larger (= older) individuals. Based on that observations, we propose that *Balloniscus sellowii* may change its anti-predator behavior along its lifetime, employing tonic immobility more often when young and small and adopting a more active escape strategy, as running, when older (= larger). In the same line, days-old workers of the ant *Solenopsis invicta* Buren responded to intraspecific aggression with tonic immobility, however when months-old the workers responded to the same aggressors with a more active response, by fighting back (Cassill et al. 2008). This strategy, employed against aggressive conspecific ants, is effective because days-old workers have a relative soft exoskeleton and at this stage tonic immobility increase their survivorship (Cassill et al. 2008).

Among the extrinsic factors that are known to influence responsiveness to tonic immobility-inducing stimuli, temperature (Miyatake et al. 2008a), light (Saxena 1967, Miyatake 2001a) and starvation (Miyatake 2001b) have been stressed. In addition to that, here we demonstrated that the type of stimulus can also influence responsiveness. Although there was no record in the literature of tonic immobility being shown by isopods upon a encounter with a predator, it can be hypothesized that the characteristic posture observed during tonic immobility by terrestrial isopods increase survivorship in two ways: it could increase resemblance with the substrate and make the animal less conspicuous to a visual predator (if compared to a moving prey) (Bergey and Weis 2006) and/or it could difficult the predator’s access to its vulnerable ventral surface. In this last scenario, tonic immobility could be effective against an attack by a small invertebrate that capture prey by biting or stinging, such as spiders or ants. By responding more to “drop” than to the other stimuli *Balloniscus glaber* seems to be responding to visual predators larger than an isopod, such as reptiles, that possibly lose their prey, in which case they would enter tonic immobility and foil the predator. On the other hand, *Balloniscus sellowii* responded more to “touch”, which could indicate a response to a smaller biting predator and the isopod would be protecting its ventral body parts. These predictions need, however, further testing.

Tonic immobility duration varies widely intraspecifically. For instance, Machado and Pomini (2008) reported that individuals of the harvestmen *Camarana flavipalpi* Soares can remain immobile for 8 s to nearly 11 min, and *Hoplobunus mexicanus* (Roewer) from 21 s to 31 min (Pomini et al. 2010). Bergey and Weis (2006) noted that fiddler crabs can remain immobile for more than two hours, but on average duration ranges from 45 to 171 s. High intraspecific variability in the duration of tonic immobility was also evidenced here, especially for *Balloniscus glaber* and *Balloniscus sellowii*. In the present study, none of the intrinsic and extrinsic factors we tested explained the variability in the species studied. The duration of response is clearly associated with prey survival. Arduino and Gould (1984) indicate that animals engaging tonic immobility could be monitoring the environment for an opportunity to escape, so the variability observed in the duration of responses could reflect the time the individuals are waiting for the risk around them to decrease. Miyatake et al. (2004) demonstrated that beetles *Tribolium castaneum* (Herbst) selected for long duration of tonic immobility were less prone to predation than those selected for short duration after exposing them to the attack of a Salticidae spider, *Hasarius adansoni* Audouin (Miyatake et al. 2004). Recent studies with insects (Miyatake et al. 2008b, Nishi et al. 2010) and spiders (Jones et al. 2011) indicate that the neurotransmitters dopamine and serotonin are involved in the regulation of tonic immobility duration.

In conclusion, we observe three different patterns of tonic immobility among the clingers studied. *Balloniscus sellowii* enters tonic immobility rarely, it is more likely to adopt an active defense, such as running. *Balloniscus glaber* is more responsive, irrespective of the stimulus applied, the sex or size of the individuals, and can stay in tonic immobility for a long time, which indicates that tonic immobility could be an important strategy for this species. *Porcellio dilatatus* is highly responsive, also irrespective of the stimulus applied, the sex or size of the individuals, but it shows a much shorter response. As pointed by Honma et al. (2006), to understand the evolutionary dynamics of prey interactions with their predators it is necessary to account for both the foraging mode of the predator and the predator-avoidance mode of the prey. Experiments with the predators of isopods are needed to investigate the effectiveness of tonic immobility and the significance of the intraspecific variability to survivorship. In the terrestrial environment, isopods can be preyed upon practically all invertebrate and vertebrate carnivores and omnivores. It seems that the multitude of anti-predator strategies they have (tonic immobility, secretion of adhesive substance, development of spiny tergites, long pereopods for running) could be a response to a history of a high predation pressure. More studies, however, are needed to investigate whether these strategies indeed improve survivorship upon encounter with a predator, and to elucidate to which predators each response works.
